# 2-(4-Bromo­phen­yl)-2-oxoethyl 4-chloro­benzoate

**DOI:** 10.1107/S1600536811022963

**Published:** 2011-06-18

**Authors:** Hoong-Kun Fun, Chin Sing Yeap, B. Garudachari, Arun M. Isloor, M. N. Satyanarayan

**Affiliations:** a X-ray Crystallography Unit, School of Physics, Universiti Sains Malaysia, 11800 USM, Penang, Malaysia; b Organic Chemistry Division, Department of Chemistry, National Institute of Technology-Karnataka, Surathkal, Mangalore 575 025, India; c Department of Physics, National Institute of Technology-Karnataka, Surathkal, Mangalore 575 025, India

## Abstract

The asymmetric unit of the title compound, C_15_H_10_BrClO_3_, consists of three crystallographically independent mol­ecules. The dihedral angles between the benzene rings in the three mol­ecules are 68.8 (2), 0.7 (3) and 66.1 (2)°. In the crystal, the three independent mol­ecules are inter­connected by C—H⋯O hydrogen bonds, leading to isolated trimers.

## Related literature

For background to phenacyl benzoate derivatives, see: Huang *et al.* (1996 ▶); Gandhi *et al.* (1995 ▶); Sheehan & Umezaw (1973 ▶); Ruzicka *et al.* (2002 ▶); Litera *et al.* (2006 ▶); Rather & Reid (1919 ▶). For related structures, see: Fun *et al.* (2011**a* ▶,*b* ▶,c* ▶). For the preparation, see: Kelly & Howard (1932 ▶).
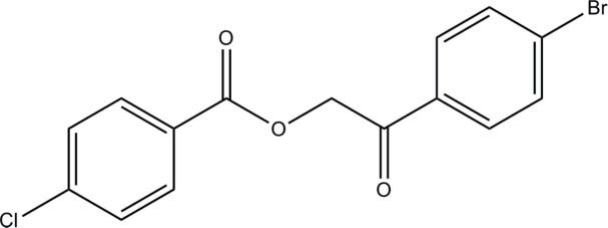


## Experimental

### 

#### Crystal data

C_15_H_10_BrClO_3_
*M* *_r_* = 353.59
Monoclinic, 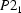

*a* = 17.1061 (11) Å
*b* = 5.3062 (4) Å
*c* = 24.0376 (16) Å
β = 101.502 (1)°
*V* = 2138.0 (3) Å^3^
*Z* = 6
Mo *K*α radiation
μ = 3.07 mm^−1^
*T* = 297 K
0.41 × 0.19 × 0.15 mm

#### Data collection

Bruker APEXII DUO CCD area-detector diffractometer
Absorption correction: multi-scan (*SADABS*; Bruker, 2009 ▶) *T*_min_ = 0.364, *T*_max_ = 0.657
18463 measured reflections
8890 independent reflections
5931 reflections with *I* > 2σ(*I*)
*R*_int_ = 0.021

#### Refinement

*R*[*F* ^2^ > 2σ(*F* ^2^)] = 0.045
*wR*(*F* ^2^) = 0.142
*S* = 1.04
8890 reflections
541 parameters
1 restraint
H-atom parameters constrained
Δρ_max_ = 0.67 e Å^−3^
Δρ_min_ = −0.54 e Å^−3^
Absolute structure: Flack (1983 ▶), 3590 Friedel pairs
Flack parameter: 0.065 (10)

### 

Data collection: *APEX2* (Bruker, 2009 ▶); cell refinement: *SAINT* (Bruker, 2009 ▶); data reduction: *SAINT*; program(s) used to solve structure: *SHELXTL* (Sheldrick, 2008 ▶); program(s) used to refine structure: *SHELXTL*; molecular graphics: *SHELXTL*; software used to prepare material for publication: *SHELXTL* and *PLATON* (Spek, 2009 ▶).

## Supplementary Material

Crystal structure: contains datablock(s) global, I. DOI: 10.1107/S1600536811022963/rz2606sup1.cif

Structure factors: contains datablock(s) I. DOI: 10.1107/S1600536811022963/rz2606Isup2.hkl

Supplementary material file. DOI: 10.1107/S1600536811022963/rz2606Isup3.cml

Additional supplementary materials:  crystallographic information; 3D view; checkCIF report

## Figures and Tables

**Table 1:** Hydrogen-bond geometry (Å, °)

*D*—H⋯*A*	*D*—H	H⋯*A*	*D*⋯*A*	*D*—H⋯*A*
C5*A*—H5*AA*⋯O3*B*	0.93	2.59	3.227 (7)	126
C5*B*—H5*BA*⋯O1*A*	0.93	2.48	3.217 (7)	136
C15*B*—H15*B*⋯O1*C*	0.93	2.59	3.254 (7)	129

**Fig. 1.**
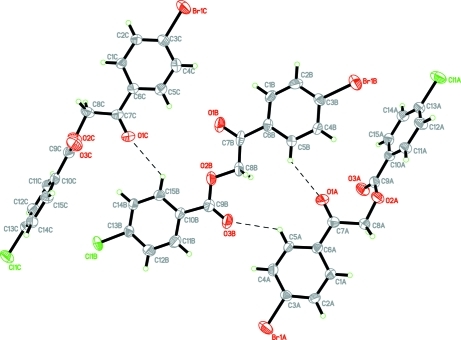
The molecular structure of the title compound, with 30% probability ellipsoids for non-H atoms. Hydrogen bonds (dashed lines) are shown.

**Fig. 2.**
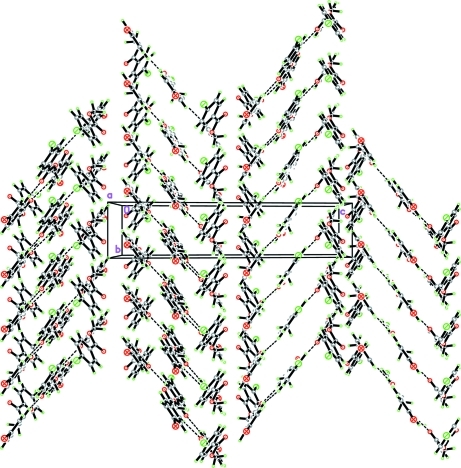
Crystal structure of title compound, showing molecules arranged into columns parallel to the b axis. Hydrogen bonds (dashed lines) are shown.

## supplementary crystallographic information

### Comment

Phenacyl benzoates derivatives are very important in identification of organic acids (Rather & Reid, 1919). These compounds undergo photolysis in neutral and mild conditions (Sheehan & Umezaw, 1973; Ruzicka *et al.*, 2002; Litera *et al.*, 2006) and find applications in the field of synthetic chemistry for the synthesis of oxazoles, imidazoles (Huang *et al.*, 1996), benzoxazepine (Gandhi *et al.*, 1995). We hereby report the crystal structure of 2-(4-bromophenyl)-2-oxoethyl 4-chlorobenzoate of potential commercial importance.

The asymmetric unit of title compound consists of three crystallographically independent molecules, *A*, *B* and *C* (Fig. 1). The dihedral angles between the two terminal phenyl rings for each molecules being 68.8 (2), 0.7 (3) and 66.1 (2)°, respectively, show that molecule *A* and *C* are in similar conformation. The geometic parameters are comparable to those previously reported structures (Fun *et al.*, 2011*a*,*b*,*c*). The three independent molecules are interconnected by C5A—H5AA···O3B, C5B—H5BA···O1A and C15B—H15B···O1C hydrogen bonds (Fig. 1, Table 1). In the crystal structure, the molecules are arranged into columns (Fig. 2) parallel to the *b* axis.

### Experimental

A mixture of 4-chlorobenzoic acid (1.0 g, 0.0063 mol) sodium carbonate (0.744 g, 0.0070 mol) and 2-bromo-1-phenylethanon (1.94 g, 0.0070 mol) in dimethylformamide (10 ml) was stirred at room temperature for 2 h. On cooling, colourless needle-shaped crystals of 2-(4-bromophenyl)-2-oxoethyl 4-chlorobenzoate separated. The compound was collected by filtration and re-crystallized from ethanol. Yield: 2.18 g, 96.88%, M. p.: 400–401 K (Kelly & Howard, 1983).

### Refinement

All hydrogen atoms were positioned geomatrically [C–H = 0.93 or 0.97 Å] and refined using a riding model, with *U*_iso_(H) = 1.2 *U*_eq_(C). The presence of pseudosymmetry in the structure suggests the higher symmetry space group *P*2_1_/*c*, but attempts to refine the structure in this space group resulted in much higher *R* and *wR* values and chemically incorrect structure. A total of 3590 Friedel pairs were used to determine the absolute structure.

### Crystal data

**Table:**

C_15_H_10_BrClO_3_	*F*(000) = 1056
*M**_r_* = 353.59	*D*_x_ = 1.648 Mg m^−^^3^
Monoclinic, *P*2_1_	Mo *K*α radiation, λ = 0.71073 Å
Hall symbol: P 2yb	Cell parameters from 4899 reflections
*a* = 17.1061 (11) Å	θ = 2.3–26.1°
*b* = 5.3062 (4) Å	µ = 3.07 mm^−^^1^
*c* = 24.0376 (16) Å	*T* = 297 K
β = 101.502 (1)°	Block, colourless
*V* = 2138.0 (3) Å^3^	0.41 × 0.19 × 0.15 mm
*Z* = 6	

### Data collection

**Table:**

Bruker APEXII DUO CCD area-detector diffractometer	8890 independent reflections
Radiation source: fine-focus sealed tube	5931 reflections with *I* > 2σ(*I*)
graphite	*R*_int_ = 0.021
φ and ω scans	θ_max_ = 27.3°, θ_min_ = 1.9°
Absorption correction: multi-scan (*SADABS*; Bruker, 2009)	*h* = −22→21
*T*_min_ = 0.364, *T*_max_ = 0.657	*k* = −6→6
18463 measured reflections	*l* = −31→31

### Refinement

**Table:**

Refinement on *F*^2^	Secondary atom site location: difference Fourier map
Least-squares matrix: full	Hydrogen site location: inferred from neighbouring sites
*R*[*F*^2^ > 2σ(*F*^2^)] = 0.045	H-atom parameters constrained
*wR*(*F*^2^) = 0.142	*w* = 1/[σ^2^(*F*_o_^2^) + (0.0741*P*)^2^ + 0.4681*P*] where *P* = (*F*_o_^2^ + 2*F*_c_^2^)/3
*S* = 1.04	(Δ/σ)_max_ = 0.001
8890 reflections	Δρ_max_ = 0.67 e Å^−^^3^
541 parameters	Δρ_min_ = −0.54 e Å^−^^3^
1 restraint	Absolute structure: Flack (1983), 3590 Friedel pairs
Primary atom site location: structure-invariant direct methods	Flack parameter: 0.065 (10)

### Special details

**Table:**

Geometry. All e.s.d.'s (except the e.s.d. in the dihedral angle between two l.s. planes) are estimated using the full covariance matrix. The cell e.s.d.'s are taken into account individually in the estimation of e.s.d.'s in distances, angles and torsion angles; correlations between e.s.d.'s in cell parameters are only used when they are defined by crystal symmetry. An approximate (isotropic) treatment of cell e.s.d.'s is used for estimating e.s.d.'s involving l.s. planes.
Refinement. Refinement of *F*^2^ against ALL reflections. The weighted *R*-factor *wR* and goodness of fit *S* are based on *F*^2^, conventional *R*-factors *R* are based on *F*, with *F* set to zero for negative *F*^2^. The threshold expression of *F*^2^ > σ(*F*^2^) is used only for calculating *R*-factors(gt) *etc*. and is not relevant to the choice of reflections for refinement. *R*-factors based on *F*^2^ are statistically about twice as large as those based on *F*, and *R*- factors based on ALL data will be even larger.

### Fractional atomic coordinates and isotropic or equivalent isotropic displacement parameters (Å^2^)

**Table:**

	*x*	*y*	*z*	*U*_iso_*/*U*_eq_	
Br1A	0.67824 (3)	1.30681 (17)	0.56131 (3)	0.0834 (2)	
Cl1A	−0.15600 (8)	0.7707 (5)	0.61298 (7)	0.0905 (6)	
O1A	0.3290 (2)	0.9241 (10)	0.63191 (17)	0.0841 (16)	
O2A	0.1988 (2)	1.1811 (8)	0.58442 (16)	0.0632 (10)	
O3A	0.1936 (2)	0.8415 (10)	0.52826 (15)	0.0736 (12)	
C1A	0.4376 (3)	1.3623 (11)	0.56106 (19)	0.0501 (12)	
H1AA	0.3959	1.4676	0.5447	0.060*	
C2A	0.5157 (3)	1.4119 (11)	0.55351 (19)	0.0526 (13)	
H2AA	0.5265	1.5531	0.5334	0.063*	
C3A	0.5759 (3)	1.2477 (12)	0.57638 (18)	0.0502 (13)	
C4A	0.5622 (3)	1.0413 (15)	0.60918 (18)	0.0556 (12)	
H4AA	0.6038	0.9348	0.6252	0.067*	
C5A	0.4865 (3)	0.9992 (11)	0.61718 (18)	0.0503 (12)	
H5AA	0.4771	0.8628	0.6392	0.060*	
C6A	0.4229 (3)	1.1541 (10)	0.59333 (17)	0.0462 (12)	
C7A	0.3413 (3)	1.0949 (12)	0.60142 (19)	0.0533 (15)	
C8A	0.2734 (3)	1.2495 (13)	0.5706 (2)	0.0622 (15)	
H8AA	0.2839	1.4259	0.5798	0.075*	
H8AB	0.2698	1.2297	0.5300	0.075*	
C9A	0.1655 (3)	0.9666 (12)	0.5602 (2)	0.0533 (15)	
C10A	0.0870 (3)	0.9183 (11)	0.57661 (18)	0.0471 (12)	
C11A	0.0551 (3)	1.0787 (13)	0.61134 (18)	0.0536 (13)	
H11A	0.0844	1.2180	0.6271	0.064*	
C12A	−0.0192 (3)	1.0367 (17)	0.6231 (2)	0.0652 (14)	
H12A	−0.0405	1.1465	0.6464	0.078*	
C13A	−0.0615 (3)	0.8289 (14)	0.5998 (2)	0.0604 (15)	
C14A	−0.0303 (3)	0.6605 (12)	0.56479 (19)	0.0569 (14)	
H14A	−0.0592	0.5200	0.5493	0.068*	
C15A	0.0445 (3)	0.7093 (13)	0.55400 (19)	0.0548 (14)	
H15A	0.0665	0.5996	0.5312	0.066*	
Br1B	0.06535 (3)	0.5564 (2)	0.73157 (2)	0.0802 (2)	
Cl1B	0.92955 (6)	−0.0329 (3)	0.77621 (5)	0.0628 (4)	
O1B	0.4359 (2)	0.0185 (11)	0.78999 (16)	0.0786 (11)	
O2B	0.54903 (19)	0.2829 (8)	0.75497 (14)	0.0627 (10)	
O3B	0.5851 (2)	0.5857 (10)	0.70158 (16)	0.0768 (12)	
C1B	0.2738 (3)	0.1656 (11)	0.7806 (2)	0.0593 (14)	
H1BA	0.2901	0.0219	0.8020	0.071*	
C2B	0.1972 (3)	0.2425 (14)	0.7741 (2)	0.0666 (17)	
H2BA	0.1620	0.1554	0.7920	0.080*	
C3B	0.1714 (3)	0.4476 (12)	0.74136 (18)	0.0525 (14)	
C4B	0.2241 (3)	0.5826 (13)	0.71591 (19)	0.0553 (14)	
H4BA	0.2066	0.7235	0.6939	0.066*	
C5B	0.3026 (3)	0.5066 (13)	0.72344 (19)	0.0552 (14)	
H5BA	0.3382	0.5967	0.7065	0.066*	
C6B	0.3285 (3)	0.2963 (12)	0.75623 (17)	0.0495 (12)	
C7B	0.4133 (3)	0.2139 (12)	0.76452 (19)	0.0579 (14)	
C8B	0.4699 (3)	0.3840 (13)	0.74273 (18)	0.0605 (16)	
H8BA	0.4527	0.4044	0.7020	0.073*	
H8BB	0.4697	0.5487	0.7602	0.073*	
C9B	0.6007 (3)	0.4055 (12)	0.7307 (2)	0.0558 (14)	
C10B	0.6816 (3)	0.2864 (12)	0.74456 (18)	0.0488 (11)	
C11B	0.7416 (3)	0.3983 (13)	0.7210 (2)	0.0677 (16)	
H11B	0.7300	0.5398	0.6980	0.081*	
C12B	0.8182 (3)	0.2997 (15)	0.7317 (2)	0.0657 (15)	
H12B	0.8586	0.3755	0.7168	0.079*	
C13B	0.8332 (3)	0.0859 (12)	0.76505 (18)	0.0497 (12)	
C14B	0.7754 (3)	−0.0248 (12)	0.78855 (18)	0.0545 (15)	
H14B	0.7873	−0.1653	0.8118	0.065*	
C15B	0.6984 (3)	0.0746 (13)	0.77740 (18)	0.0525 (13)	
H15B	0.6584	−0.0031	0.7923	0.063*	
Br1C	0.32100 (3)	−0.72787 (16)	0.94037 (3)	0.0849 (3)	
Cl1C	1.15846 (7)	−0.2313 (4)	0.89129 (6)	0.0805 (5)	
O1C	0.6700 (2)	−0.3571 (9)	0.86746 (17)	0.0800 (14)	
O2C	0.7989 (2)	−0.6225 (9)	0.91351 (17)	0.0693 (12)	
O3C	0.8057 (2)	−0.2890 (10)	0.97126 (16)	0.0758 (12)	
C1C	0.5592 (3)	−0.7933 (11)	0.93756 (19)	0.0515 (13)	
H1CA	0.5997	−0.9063	0.9520	0.062*	
C2C	0.4832 (3)	−0.8341 (12)	0.9467 (2)	0.0552 (14)	
H2CA	0.4729	−0.9727	0.9678	0.066*	
C3C	0.4234 (3)	−0.6727 (13)	0.92500 (19)	0.0533 (13)	
C4C	0.4372 (3)	−0.4662 (16)	0.89371 (19)	0.0611 (14)	
H4CA	0.3958	−0.3569	0.8788	0.073*	
C5C	0.5134 (3)	−0.4232 (13)	0.88469 (18)	0.0549 (13)	
H5CA	0.5231	−0.2843	0.8635	0.066*	
C6C	0.5757 (3)	−0.5854 (10)	0.90700 (18)	0.0450 (12)	
C7C	0.6575 (3)	−0.5282 (11)	0.8977 (2)	0.0519 (14)	
C8C	0.7228 (3)	−0.6899 (14)	0.9273 (2)	0.0642 (15)	
H8CA	0.7264	−0.6749	0.9679	0.077*	
H8CB	0.7108	−0.8643	0.9168	0.077*	
C9C	0.8341 (3)	−0.4191 (13)	0.93900 (19)	0.0511 (13)	
C10C	0.9124 (3)	−0.3701 (10)	0.92392 (18)	0.0482 (13)	
C11C	0.9457 (3)	−0.5376 (11)	0.89043 (19)	0.0517 (13)	
H11C	0.9170	−0.6783	0.8748	0.062*	
C12C	1.0218 (3)	−0.4933 (14)	0.8805 (2)	0.0573 (14)	
H12C	1.0445	−0.6050	0.8585	0.069*	
C13C	1.0634 (3)	−0.2850 (12)	0.9034 (2)	0.0514 (13)	
C14C	1.0313 (3)	−0.1212 (12)	0.9361 (2)	0.0566 (13)	
H14C	1.0605	0.0188	0.9516	0.068*	
C15C	0.9556 (3)	−0.1613 (13)	0.9465 (2)	0.0542 (13)	
H15C	0.9339	−0.0477	0.9687	0.065*	

### Atomic displacement parameters (Å^2^)

**Table:**

	*U*^11^	*U*^22^	*U*^33^	*U*^12^	*U*^13^	*U*^23^
Br1A	0.0459 (3)	0.1128 (7)	0.0917 (4)	−0.0160 (4)	0.0140 (3)	0.0005 (4)
Cl1A	0.0560 (7)	0.1171 (17)	0.1044 (11)	−0.0214 (11)	0.0302 (8)	0.0042 (12)
O1A	0.065 (2)	0.102 (4)	0.087 (3)	−0.003 (2)	0.019 (2)	0.047 (3)
O2A	0.0488 (19)	0.057 (3)	0.090 (2)	−0.005 (2)	0.0276 (18)	−0.009 (2)
O3A	0.065 (2)	0.082 (3)	0.082 (2)	0.003 (3)	0.0341 (19)	−0.015 (3)
C1A	0.050 (2)	0.043 (3)	0.057 (3)	0.000 (3)	0.008 (2)	0.003 (3)
C2A	0.058 (3)	0.045 (3)	0.057 (3)	−0.004 (3)	0.017 (2)	0.009 (2)
C3A	0.043 (2)	0.056 (4)	0.048 (2)	−0.009 (3)	0.003 (2)	−0.003 (3)
C4A	0.050 (2)	0.053 (3)	0.060 (3)	0.004 (4)	0.000 (2)	0.004 (3)
C5A	0.053 (3)	0.041 (3)	0.054 (2)	0.003 (3)	0.004 (2)	0.013 (3)
C6A	0.050 (3)	0.044 (3)	0.042 (2)	−0.004 (2)	0.004 (2)	0.004 (2)
C7A	0.052 (3)	0.058 (4)	0.049 (2)	−0.008 (3)	0.010 (2)	0.001 (3)
C8A	0.050 (3)	0.054 (4)	0.087 (3)	0.004 (3)	0.024 (3)	0.012 (3)
C9A	0.048 (3)	0.055 (4)	0.057 (3)	0.008 (3)	0.011 (2)	0.003 (3)
C10A	0.044 (2)	0.049 (3)	0.048 (2)	−0.003 (2)	0.007 (2)	0.002 (2)
C11A	0.053 (3)	0.048 (3)	0.062 (3)	−0.007 (3)	0.018 (2)	−0.009 (3)
C12A	0.062 (3)	0.069 (4)	0.071 (3)	0.000 (4)	0.029 (2)	−0.011 (4)
C13A	0.046 (3)	0.071 (4)	0.065 (3)	0.001 (3)	0.011 (2)	0.015 (3)
C14A	0.052 (3)	0.057 (4)	0.058 (3)	−0.014 (3)	0.001 (2)	0.000 (3)
C15A	0.060 (3)	0.055 (4)	0.050 (3)	0.010 (3)	0.012 (2)	−0.002 (3)
Br1B	0.0535 (3)	0.1015 (6)	0.0861 (4)	0.0141 (4)	0.0153 (3)	−0.0053 (4)
Cl1B	0.0359 (5)	0.0806 (12)	0.0704 (7)	0.0202 (6)	0.0067 (5)	−0.0083 (7)
O1B	0.072 (2)	0.070 (3)	0.091 (2)	0.030 (3)	0.0089 (19)	0.019 (3)
O2B	0.0516 (18)	0.073 (3)	0.0619 (19)	0.024 (2)	0.0083 (15)	0.008 (2)
O3B	0.073 (2)	0.072 (3)	0.085 (2)	0.028 (3)	0.0124 (19)	0.023 (3)
C1B	0.073 (3)	0.045 (3)	0.062 (3)	0.013 (3)	0.018 (3)	0.016 (3)
C2B	0.076 (4)	0.064 (4)	0.064 (3)	−0.008 (3)	0.026 (3)	0.016 (3)
C3B	0.044 (2)	0.068 (4)	0.046 (2)	0.003 (2)	0.0098 (19)	−0.006 (3)
C4B	0.053 (3)	0.049 (4)	0.062 (3)	0.012 (3)	0.009 (2)	0.015 (3)
C5B	0.044 (2)	0.058 (4)	0.062 (3)	0.007 (3)	0.008 (2)	0.015 (3)
C6B	0.055 (3)	0.050 (3)	0.040 (2)	0.012 (3)	0.0021 (19)	−0.002 (2)
C7B	0.068 (3)	0.051 (4)	0.048 (3)	0.029 (3)	−0.005 (2)	−0.001 (2)
C8B	0.057 (3)	0.079 (4)	0.045 (2)	0.030 (3)	0.007 (2)	0.006 (2)
C9B	0.062 (3)	0.061 (4)	0.046 (2)	0.014 (3)	0.015 (2)	0.000 (3)
C10B	0.055 (2)	0.047 (3)	0.044 (2)	0.011 (3)	0.009 (2)	0.007 (2)
C11B	0.077 (3)	0.065 (4)	0.065 (3)	0.014 (3)	0.021 (3)	0.016 (3)
C12B	0.057 (3)	0.074 (4)	0.073 (3)	0.005 (3)	0.030 (2)	0.006 (3)
C13B	0.050 (2)	0.046 (3)	0.053 (2)	0.012 (3)	0.010 (2)	0.001 (3)
C14B	0.050 (3)	0.059 (4)	0.054 (2)	0.012 (3)	0.008 (2)	0.014 (3)
C15B	0.053 (3)	0.054 (4)	0.053 (2)	0.003 (3)	0.017 (2)	0.005 (3)
Br1C	0.0474 (3)	0.1115 (7)	0.0968 (4)	−0.0085 (4)	0.0169 (3)	−0.0075 (4)
Cl1C	0.0425 (6)	0.1100 (15)	0.0921 (9)	−0.0088 (9)	0.0205 (6)	0.0061 (10)
O1C	0.066 (2)	0.087 (4)	0.090 (3)	−0.011 (2)	0.022 (2)	0.036 (3)
O2C	0.055 (2)	0.064 (3)	0.096 (3)	−0.001 (2)	0.034 (2)	−0.004 (3)
O3C	0.065 (2)	0.086 (3)	0.082 (2)	−0.006 (2)	0.0283 (19)	−0.024 (3)
C1C	0.054 (3)	0.044 (3)	0.059 (3)	0.006 (3)	0.017 (2)	0.010 (3)
C2C	0.052 (3)	0.053 (4)	0.060 (3)	−0.007 (3)	0.012 (2)	0.007 (3)
C3C	0.047 (2)	0.057 (4)	0.057 (3)	−0.005 (3)	0.012 (2)	−0.010 (3)
C4C	0.052 (3)	0.064 (4)	0.062 (3)	0.009 (4)	−0.002 (2)	−0.001 (4)
C5C	0.064 (3)	0.048 (4)	0.051 (2)	−0.008 (3)	0.006 (2)	−0.002 (3)
C6C	0.052 (3)	0.038 (3)	0.046 (2)	−0.002 (2)	0.013 (2)	−0.005 (2)
C7C	0.057 (3)	0.046 (4)	0.056 (3)	−0.008 (3)	0.018 (2)	0.004 (3)
C8C	0.052 (3)	0.056 (4)	0.089 (4)	−0.012 (3)	0.024 (3)	−0.003 (3)
C9C	0.051 (3)	0.050 (4)	0.052 (2)	0.002 (3)	0.011 (2)	0.001 (3)
C10C	0.049 (3)	0.048 (4)	0.047 (2)	0.012 (2)	0.007 (2)	0.005 (2)
C11C	0.052 (3)	0.048 (4)	0.057 (2)	0.004 (3)	0.016 (2)	0.004 (2)
C12C	0.055 (3)	0.059 (4)	0.063 (3)	0.006 (3)	0.022 (2)	0.005 (3)
C13C	0.041 (2)	0.058 (4)	0.056 (3)	0.002 (3)	0.012 (2)	0.009 (3)
C14C	0.057 (3)	0.049 (3)	0.061 (3)	−0.004 (3)	0.004 (2)	0.001 (3)
C15C	0.057 (3)	0.049 (4)	0.057 (3)	0.000 (3)	0.012 (2)	−0.001 (3)

### Geometric parameters (Å, °)

**Table:**

Br1A—C3A	1.884 (4)	C6B—C7B	1.488 (7)
Cl1A—C13A	1.735 (5)	C7B—C8B	1.493 (9)
O1A—C7A	1.210 (7)	C8B—H8BA	0.9700
O2A—C9A	1.351 (7)	C8B—H8BB	0.9700
O2A—C8A	1.428 (6)	C9B—C10B	1.497 (7)
O3A—C9A	1.187 (6)	C10B—C15B	1.370 (8)
C1A—C6A	1.401 (7)	C10B—C11B	1.401 (7)
C1A—C2A	1.409 (6)	C11B—C12B	1.386 (8)
C1A—H1AA	0.9300	C11B—H11B	0.9300
C2A—C3A	1.376 (7)	C12B—C13B	1.384 (9)
C2A—H2AA	0.9300	C12B—H12B	0.9300
C3A—C4A	1.396 (9)	C13B—C14B	1.365 (6)
C4A—C5A	1.366 (6)	C14B—C15B	1.395 (6)
C4A—H4AA	0.9300	C14B—H14B	0.9300
C5A—C6A	1.392 (7)	C15B—H15B	0.9300
C5A—H5AA	0.9300	Br1C—C3C	1.884 (4)
C6A—C7A	1.481 (6)	Cl1C—C13C	1.732 (5)
C7A—C8A	1.493 (7)	O1C—C7C	1.208 (6)
C8A—H8AA	0.9700	O2C—C9C	1.325 (8)
C8A—H8AB	0.9700	O2C—C8C	1.451 (5)
C9A—C10A	1.495 (6)	O3C—C9C	1.210 (6)
C10A—C15A	1.377 (8)	C1C—C2C	1.378 (6)
C10A—C11A	1.379 (7)	C1C—C6C	1.386 (7)
C11A—C12A	1.375 (6)	C1C—H1CA	0.9300
C11A—H11A	0.9300	C2C—C3C	1.356 (8)
C12A—C13A	1.375 (10)	C2C—H2CA	0.9300
C12A—H12A	0.9300	C3C—C4C	1.376 (10)
C13A—C14A	1.405 (8)	C4C—C5C	1.382 (6)
C14A—C15A	1.379 (7)	C4C—H4CA	0.9300
C14A—H14A	0.9300	C5C—C6C	1.392 (7)
C15A—H15A	0.9300	C5C—H5CA	0.9300
Br1B—C3B	1.873 (5)	C6C—C7C	1.491 (6)
Cl1B—C13B	1.735 (5)	C7C—C8C	1.474 (8)
O1B—C7B	1.227 (8)	C8C—H8CA	0.9700
O2B—C9B	1.324 (7)	C8C—H8CB	0.9700
O2B—C8B	1.431 (5)	C9C—C10C	1.477 (6)
O3B—C9B	1.183 (7)	C10C—C15C	1.382 (8)
C1B—C2B	1.351 (7)	C10C—C11C	1.395 (7)
C1B—C6B	1.387 (7)	C11C—C12C	1.390 (6)
C1B—H1BA	0.9300	C11C—H11C	0.9300
C2B—C3B	1.364 (9)	C12C—C13C	1.368 (9)
C2B—H2BA	0.9300	C12C—H12C	0.9300
C3B—C4B	1.385 (7)	C13C—C14C	1.358 (7)
C4B—C5B	1.379 (6)	C14C—C15C	1.383 (7)
C4B—H4BA	0.9300	C14C—H14C	0.9300
C5B—C6B	1.387 (8)	C15C—H15C	0.9300
C5B—H5BA	0.9300		
			
C9A—O2A—C8A	115.7 (5)	O2B—C8B—H8BB	109.5
C6A—C1A—C2A	119.9 (5)	C7B—C8B—H8BB	109.5
C6A—C1A—H1AA	120.1	H8BA—C8B—H8BB	108.1
C2A—C1A—H1AA	120.1	O3B—C9B—O2B	124.5 (5)
C3A—C2A—C1A	118.9 (5)	O3B—C9B—C10B	124.2 (5)
C3A—C2A—H2AA	120.6	O2B—C9B—C10B	111.2 (5)
C1A—C2A—H2AA	120.6	C15B—C10B—C11B	119.6 (5)
C2A—C3A—C4A	121.8 (4)	C15B—C10B—C9B	123.6 (5)
C2A—C3A—Br1A	117.9 (4)	C11B—C10B—C9B	116.8 (5)
C4A—C3A—Br1A	120.4 (4)	C12B—C11B—C10B	120.5 (6)
C5A—C4A—C3A	118.6 (5)	C12B—C11B—H11B	119.8
C5A—C4A—H4AA	120.7	C10B—C11B—H11B	119.8
C3A—C4A—H4AA	120.7	C13B—C12B—C11B	118.4 (5)
C4A—C5A—C6A	122.0 (5)	C13B—C12B—H12B	120.8
C4A—C5A—H5AA	119.0	C11B—C12B—H12B	120.8
C6A—C5A—H5AA	119.0	C14B—C13B—C12B	121.8 (5)
C5A—C6A—C1A	118.8 (4)	C14B—C13B—Cl1B	121.6 (4)
C5A—C6A—C7A	120.0 (5)	C12B—C13B—Cl1B	116.6 (4)
C1A—C6A—C7A	121.2 (5)	C13B—C14B—C15B	119.5 (5)
O1A—C7A—C6A	121.4 (5)	C13B—C14B—H14B	120.3
O1A—C7A—C8A	120.0 (4)	C15B—C14B—H14B	120.3
C6A—C7A—C8A	118.6 (5)	C10B—C15B—C14B	120.2 (5)
O2A—C8A—C7A	112.8 (5)	C10B—C15B—H15B	119.9
O2A—C8A—H8AA	109.0	C14B—C15B—H15B	119.9
C7A—C8A—H8AA	109.0	C9C—O2C—C8C	116.2 (4)
O2A—C8A—H8AB	109.0	C2C—C1C—C6C	120.6 (5)
C7A—C8A—H8AB	109.0	C2C—C1C—H1CA	119.7
H8AA—C8A—H8AB	107.8	C6C—C1C—H1CA	119.7
O3A—C9A—O2A	124.0 (5)	C3C—C2C—C1C	120.2 (5)
O3A—C9A—C10A	125.1 (5)	C3C—C2C—H2CA	119.9
O2A—C9A—C10A	110.8 (5)	C1C—C2C—H2CA	119.9
C15A—C10A—C11A	119.6 (4)	C2C—C3C—C4C	121.0 (4)
C15A—C10A—C9A	117.7 (5)	C2C—C3C—Br1C	119.1 (4)
C11A—C10A—C9A	122.6 (5)	C4C—C3C—Br1C	119.9 (4)
C12A—C11A—C10A	121.2 (6)	C3C—C4C—C5C	119.1 (6)
C12A—C11A—H11A	119.4	C3C—C4C—H4CA	120.4
C10A—C11A—H11A	119.4	C5C—C4C—H4CA	120.4
C11A—C12A—C13A	118.7 (6)	C4C—C5C—C6C	120.8 (6)
C11A—C12A—H12A	120.7	C4C—C5C—H5CA	119.6
C13A—C12A—H12A	120.7	C6C—C5C—H5CA	119.6
C12A—C13A—C14A	121.4 (4)	C1C—C6C—C5C	118.4 (4)
C12A—C13A—Cl1A	120.1 (5)	C1C—C6C—C7C	122.6 (5)
C14A—C13A—Cl1A	118.4 (5)	C5C—C6C—C7C	119.0 (5)
C15A—C14A—C13A	118.1 (5)	O1C—C7C—C8C	121.3 (5)
C15A—C14A—H14A	120.9	O1C—C7C—C6C	121.8 (5)
C13A—C14A—H14A	120.9	C8C—C7C—C6C	116.9 (4)
C10A—C15A—C14A	120.9 (5)	O2C—C8C—C7C	112.0 (5)
C10A—C15A—H15A	119.6	O2C—C8C—H8CA	109.2
C14A—C15A—H15A	119.6	C7C—C8C—H8CA	109.2
C9B—O2B—C8B	114.3 (5)	O2C—C8C—H8CB	109.2
C2B—C1B—C6B	121.4 (5)	C7C—C8C—H8CB	109.2
C2B—C1B—H1BA	119.3	H8CA—C8C—H8CB	107.9
C6B—C1B—H1BA	119.3	O3C—C9C—O2C	123.9 (5)
C1B—C2B—C3B	120.2 (5)	O3C—C9C—C10C	123.8 (6)
C1B—C2B—H2BA	119.9	O2C—C9C—C10C	112.3 (5)
C3B—C2B—H2BA	119.9	C15C—C10C—C11C	119.3 (4)
C2B—C3B—C4B	120.2 (5)	C15C—C10C—C9C	119.0 (5)
C2B—C3B—Br1B	120.8 (4)	C11C—C10C—C9C	121.6 (5)
C4B—C3B—Br1B	119.0 (4)	C12C—C11C—C10C	119.7 (5)
C5B—C4B—C3B	119.5 (6)	C12C—C11C—H11C	120.2
C5B—C4B—H4BA	120.2	C10C—C11C—H11C	120.2
C3B—C4B—H4BA	120.2	C13C—C12C—C11C	119.8 (5)
C4B—C5B—C6B	120.2 (5)	C13C—C12C—H12C	120.1
C4B—C5B—H5BA	119.9	C11C—C12C—H12C	120.1
C6B—C5B—H5BA	119.9	C14C—C13C—C12C	120.8 (5)
C1B—C6B—C5B	118.4 (4)	C14C—C13C—Cl1C	119.5 (5)
C1B—C6B—C7B	121.2 (5)	C12C—C13C—Cl1C	119.7 (4)
C5B—C6B—C7B	120.4 (5)	C13C—C14C—C15C	120.4 (5)
O1B—C7B—C6B	121.1 (6)	C13C—C14C—H14C	119.8
O1B—C7B—C8B	121.6 (5)	C15C—C14C—H14C	119.8
C6B—C7B—C8B	117.2 (5)	C10C—C15C—C14C	120.0 (5)
O2B—C8B—C7B	110.8 (5)	C10C—C15C—H15C	120.0
O2B—C8B—H8BA	109.5	C14C—C15C—H15C	120.0
C7B—C8B—H8BA	109.5		
			
C6A—C1A—C2A—C3A	2.5 (7)	C8B—O2B—C9B—O3B	0.8 (8)
C1A—C2A—C3A—C4A	−3.3 (8)	C8B—O2B—C9B—C10B	−179.0 (4)
C1A—C2A—C3A—Br1A	175.9 (4)	O3B—C9B—C10B—C15B	−178.7 (6)
C2A—C3A—C4A—C5A	1.8 (8)	O2B—C9B—C10B—C15B	1.2 (7)
Br1A—C3A—C4A—C5A	−177.3 (4)	O3B—C9B—C10B—C11B	−0.2 (8)
C3A—C4A—C5A—C6A	0.5 (8)	O2B—C9B—C10B—C11B	179.6 (5)
C4A—C5A—C6A—C1A	−1.3 (8)	C15B—C10B—C11B—C12B	−1.5 (9)
C4A—C5A—C6A—C7A	177.7 (5)	C9B—C10B—C11B—C12B	−180.0 (5)
C2A—C1A—C6A—C5A	−0.2 (7)	C10B—C11B—C12B—C13B	1.4 (9)
C2A—C1A—C6A—C7A	−179.2 (5)	C11B—C12B—C13B—C14B	−1.7 (9)
C5A—C6A—C7A—O1A	5.4 (8)	C11B—C12B—C13B—Cl1B	179.0 (5)
C1A—C6A—C7A—O1A	−175.7 (5)	C12B—C13B—C14B—C15B	2.0 (8)
C5A—C6A—C7A—C8A	−174.0 (5)	Cl1B—C13B—C14B—C15B	−178.7 (4)
C1A—C6A—C7A—C8A	4.9 (7)	C11B—C10B—C15B—C14B	1.8 (8)
C9A—O2A—C8A—C7A	−76.3 (6)	C9B—C10B—C15B—C14B	−179.8 (5)
O1A—C7A—C8A—O2A	3.6 (8)	C13B—C14B—C15B—C10B	−2.0 (8)
C6A—C7A—C8A—O2A	−177.0 (5)	C6C—C1C—C2C—C3C	1.0 (8)
C8A—O2A—C9A—O3A	−1.7 (7)	C1C—C2C—C3C—C4C	−0.1 (8)
C8A—O2A—C9A—C10A	−178.6 (4)	C1C—C2C—C3C—Br1C	−178.0 (4)
O3A—C9A—C10A—C15A	1.5 (8)	C2C—C3C—C4C—C5C	−0.3 (8)
O2A—C9A—C10A—C15A	178.3 (5)	Br1C—C3C—C4C—C5C	177.5 (4)
O3A—C9A—C10A—C11A	−176.0 (5)	C3C—C4C—C5C—C6C	−0.2 (8)
O2A—C9A—C10A—C11A	0.8 (7)	C2C—C1C—C6C—C5C	−1.6 (7)
C15A—C10A—C11A—C12A	−1.2 (8)	C2C—C1C—C6C—C7C	177.8 (5)
C9A—C10A—C11A—C12A	176.3 (5)	C4C—C5C—C6C—C1C	1.2 (7)
C10A—C11A—C12A—C13A	0.5 (9)	C4C—C5C—C6C—C7C	−178.3 (5)
C11A—C12A—C13A—C14A	0.2 (9)	C1C—C6C—C7C—O1C	174.4 (5)
C11A—C12A—C13A—Cl1A	−179.3 (5)	C5C—C6C—C7C—O1C	−6.2 (7)
C12A—C13A—C14A—C15A	−0.2 (8)	C1C—C6C—C7C—C8C	−5.8 (7)
Cl1A—C13A—C14A—C15A	179.3 (4)	C5C—C6C—C7C—C8C	173.6 (5)
C11A—C10A—C15A—C14A	1.2 (8)	C9C—O2C—C8C—C7C	77.1 (6)
C9A—C10A—C15A—C14A	−176.4 (5)	O1C—C7C—C8C—O2C	−2.3 (8)
C13A—C14A—C15A—C10A	−0.5 (8)	C6C—C7C—C8C—O2C	178.0 (4)
C6B—C1B—C2B—C3B	2.3 (9)	C8C—O2C—C9C—O3C	−1.4 (8)
C1B—C2B—C3B—C4B	−1.9 (9)	C8C—O2C—C9C—C10C	178.1 (4)
C1B—C2B—C3B—Br1B	179.8 (5)	O3C—C9C—C10C—C15C	−1.6 (8)
C2B—C3B—C4B—C5B	0.7 (8)	O2C—C9C—C10C—C15C	178.9 (5)
Br1B—C3B—C4B—C5B	179.1 (4)	O3C—C9C—C10C—C11C	174.3 (5)
C3B—C4B—C5B—C6B	0.1 (8)	O2C—C9C—C10C—C11C	−5.2 (7)
C2B—C1B—C6B—C5B	−1.5 (8)	C15C—C10C—C11C—C12C	0.4 (7)
C2B—C1B—C6B—C7B	178.7 (5)	C9C—C10C—C11C—C12C	−175.5 (5)
C4B—C5B—C6B—C1B	0.3 (8)	C10C—C11C—C12C—C13C	−0.5 (8)
C4B—C5B—C6B—C7B	−179.9 (5)	C11C—C12C—C13C—C14C	0.5 (8)
C1B—C6B—C7B—O1B	4.9 (8)	C11C—C12C—C13C—Cl1C	179.6 (4)
C5B—C6B—C7B—O1B	−174.8 (5)	C12C—C13C—C14C—C15C	−0.6 (8)
C1B—C6B—C7B—C8B	−172.6 (5)	Cl1C—C13C—C14C—C15C	−179.7 (4)
C5B—C6B—C7B—C8B	7.7 (7)	C11C—C10C—C15C—C14C	−0.4 (7)
C9B—O2B—C8B—C7B	171.7 (4)	C9C—C10C—C15C—C14C	175.6 (5)
O1B—C7B—C8B—O2B	1.1 (7)	C13C—C14C—C15C—C10C	0.5 (8)
C6B—C7B—C8B—O2B	178.6 (4)		

### Hydrogen-bond geometry (Å, °)

**Table:**

*D*—H···*A*	*D*—H	H···*A*	*D*···*A*	*D*—H···*A*
C5A—H5AA···O3B	0.93	2.59	3.227 (7)	126
C5B—H5BA···O1A	0.93	2.48	3.217 (7)	136
C15B—H15B···O1C	0.93	2.59	3.254 (7)	129
